# Wickersheimer’s Method of Preserving Anatomical Preparations and Whole Bodies

**Published:** 1880-02

**Authors:** F. C. Hotz

**Affiliations:** Chicago


### ARTICLE XI.

### WICKERSHEIMER'S METHOD OF PRESERVING ANATOMICAL PREPARATIONS AND WHOLE BODIES.

CHICAGO, Jan. 20, 1880.

*Editors of the* CHICAGO MEDICAL JOURNAL AND EXAMINER:

About a year ago I read in a German newspaper that Dr. Wickersheimer, prosector in the anatomical institute at the University of Berlin, had invented a new preserving fluid, by which it was possible to preserve any organic bodies (plants, animals and human bodies) so perfectly that they retained their original natural form, color and limpid texture. The inventor, at first, secured a patent for his invention, but in the interest of science he relinquished it; and everybody may employ Wickersheimer's method, which is thus described in the *Deutsche Reichsanzeiger:*

"The preserving fluid is prepared as follows: 100 grams of alum, 25 grams of cooking salt, 12 grams of saltpetre, 60 grams of potash and 10 grams of arsenious acid are dissolved in 3000 grams of boiling water. The solution is cooled and filtered. To every 10 liters of this neutral, colorless and oderless fluid there are added 4 liters of glycerine and one liter of methyl alcohol. For the dry preservation the preparations are immersed in the fluid from six to twelve days, according to their size, and then dried in the air. Organs, like the lungs and intestines, are filled with the fluid before immersion, and blown up before drying. Small animals and portions of bodies, which are to be preserved in their natural colors, are simply kept immersed in the fluid. For embalming, the body is injected and laid in the fluid for several days; it is then taken out, rubbed off and dried, and wrapped in linen or oil-cloth saturated with the fluid, and encased in air-tight receptacles. If bodies are to be temporarily embalmed for scientific purposes the injection of 1½ liters (for children) to 5 liters (for adults) is sufficient. Even after years, the cut surface of the muscles shows the appearance of those of a fresh corpse. Bodies treated in this way have not the least odor."

As this invention seems to be of the utmost importance for anatomy and consequently will be of great interest to the medical profession, you may perhaps deem this brief note worthy of publication. Respectfully, F. C. HOTZ, M.D.

